# Invasive and non-invasive assessment of ischaemia in chronic coronary syndromes: translating pathophysiology to clinical practice

**DOI:** 10.1093/eurheartj/ehab548

**Published:** 2021-09-13

**Authors:** Ozan M Demir, Haseeb Rahman, Tim P van de Hoef, Javier Escaned, Jan J Piek, Sven Plein, Divaka Perera

**Affiliations:** British Heart Foundation Centre of Excellence and National Institute for Health Research Biomedical Research Centre at the School of Cardiovascular Medicine and Sciences, King’s College London, Westminster Bridge Road, London SE1 7EH, UK; British Heart Foundation Centre of Excellence and National Institute for Health Research Biomedical Research Centre at the School of Cardiovascular Medicine and Sciences, King’s College London, Westminster Bridge Road, London SE1 7EH, UK; Department of Clinical and Experimental Cardiology, Amsterdam UMC, University of Amsterdam, Meibergdreef 9, Amsterdam 1105 AZ, Netherlands; Department of Cardiology, Hospital Clínico San Carlos IDISCC, Complutense University of Madrid, SpainCalle del Prof Martín Lagos, Madrid 28040, Spain; Department of Clinical and Experimental Cardiology, Amsterdam UMC, University of Amsterdam, Meibergdreef 9, Amsterdam 1105 AZ, Netherlands; Department of Biomedical Imaging Science, Leeds Institute of Cardiovascular and Metabolic Medicine, University of Leeds, Leeds LS2 9JT, UK; British Heart Foundation Centre of Excellence and National Institute for Health Research Biomedical Research Centre at the School of Cardiovascular Medicine and Sciences, King’s College London, Westminster Bridge Road, London SE1 7EH, UK

**Keywords:** Coronary physiology, Coronary circulation, Stable coronary artery disease

## Abstract

Intracoronary physiology testing has emerged as a valuable diagnostic approach in the management of patients with chronic coronary syndrome, circumventing limitations like inferring coronary function from anatomical assessment and low spatial resolution associated with angiography or non-invasive tests. The value of hyperaemic translesional pressure ratios to estimate the functional relevance of coronary stenoses is supported by a wealth of prognostic data. The continuing drive to further simplify this approach led to the development of non-hyperaemic pressure-based indices. Recent attention has focussed on estimating physiology without even measuring coronary pressure. However, the reduction in procedural time and ease of accessibility afforded by these simplifications needs to be counterbalanced against the increasing burden of physiological assumptions, which may impact on the ability to reliably identify an ischaemic substrate, the ultimate goal during catheter laboratory assessment. In that regard, measurement of both coronary pressure and flow enables comprehensive physiological evaluation of both epicardial and microcirculatory components of the vasculature, although widespread adoption has been hampered by perceived technical complexity and, in general, an underappreciation of the role of the microvasculature. In parallel, entirely non-invasive tools have matured, with the utilization of various techniques including computational fluid dynamic and quantitative perfusion analysis. This review article appraises the strengths and limitations for each test in investigating myocardial ischaemia and discusses a comprehensive algorithm that could be used to obtain a diagnosis in all patients with angina scheduled for coronary angiography, including those who are not found to have obstructive epicardial coronary disease.

## Graphical Abstract

**Graphical Abstract:**
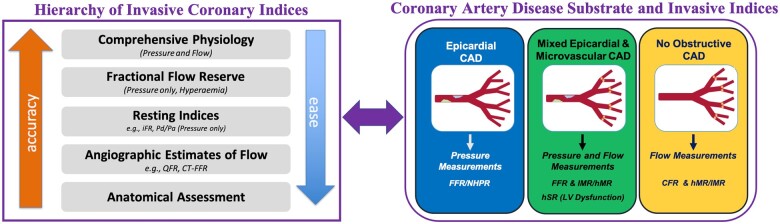
Illustration of hierarchy of coronary indices and optimal coronary indices by coronary artery disease substrate. CFR, coronary flow reserve; CT, computed tomography; FFR, fractional flow reserve; hMR, hyperaemic microvascular resistance; hSR, hyperaemic stenosis resistance; iFR, instantaneous wave-free ratio; IMR, index of microvascular resistance; NHPR, non-hyperaemic pressure ratio; Pd/Pa, resting distal to aortic pressure ratio; QFR, quantitative flow reserve.

## Introduction

**Figure 1:**
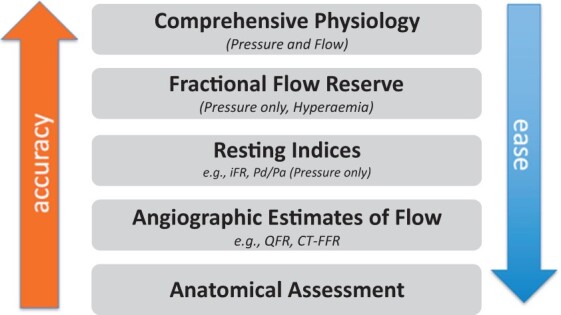
Illustration of inverse relationship between accuracy and ease of use for physiological indices of coronary circulatory evaluation. CT, computed tomography; FFR, fractional flow reserve; iFR, instantaneous wave-free ratio; Pd/Pa, resting distal to aortic pressure ratio; QFR, quantitative flow reserve.

For decades, coronary angiography served as the gold standard in the diagnosis of coronary artery disease (CAD), with a supportive role for non-invasive tests in clinical decision-making. Yet, following a call in the latter part of the 20th century,^1^ for greater reliance on physiology and less on anatomy alone, there has been a growing move to functionally characterize the coronary circulation, enabled by a new armamentarium of coronary physiology tools. The demonstration of the clinical and economic benefits of strategies based on functional coronary and myocardial assessment has resulted in an increase in physiology-guided management of patients with CAD, but uptake still lags behind the evidence base.^2–4^ The coronary circulation comprises distinct anatomical and functional compartments, working in concert to match blood supply to highly varying myocardial oxygen requirements. During increased periods of demand, such as exercise, increased aortic pressure, reduced microvascular resistance, and augmentation of the dynamic interaction between the contracting heart and vasculature (cardiac‒coronary coupling) accentuate coronary blood flow to ensure adequate transmural perfusion of the left ventricle. Simultaneous measurement of distal coronary pressure and flow, across a range of physiological conditions, allows comprehensive characterization of the epicardial and microvascular compartments. However, whilst the advent of ultra-thin sensor-tipped guidewires allows these measurements to be carried out in a clinical setting, the measurement of coronary blood flow still remains challenging and often time-consuming. Instead, the observation that the pressure drop across an epicardial artery stenosis is related to blood flow across the stenosis and the fact that pressure is (more or less) linearly related to flow in certain conditions has led to the emergence of techniques to assess stenosis significance based on pressure measurements alone. The most commonly studied catheter laboratory tool is the pressure-derived index, fractional flow reserve (FFR), the use of which is supported by a wealth of prognostic data.^5^ Further iterative simplifications of pressure-based epicardial artery assessment have occurred in recent years, first with techniques that have abandoned the need to induce hyperaemia during pressure measurements and more recently, angiographic techniques that obviate the need to measure intracoronary pressure at all. In parallel, entirely non-invasive tools have matured, with the utilization of various techniques including computational fluid dynamic modelling-based computed tomography-derived FFR (CT-FFR) and perfusion imaging, in particular with positron emission tomography (PET) and cardiovascular magnetic resonance (CMR). These methods can complement or, in some patients, be performed instead of invasive physiological measurements. In this review, we cover the physiological principles underlying the regulation of coronary blood flow in health and disease states and explore the potential trade-off between ease and accuracy, as increasing assumptions are applied when moving from comprehensive pressure and flow to the iterative simplifications (*Graphical Abstract* and *Figure 1*).

## Measuring coronary flow in the cardiac catheterization laboratory

**Figure 2:**
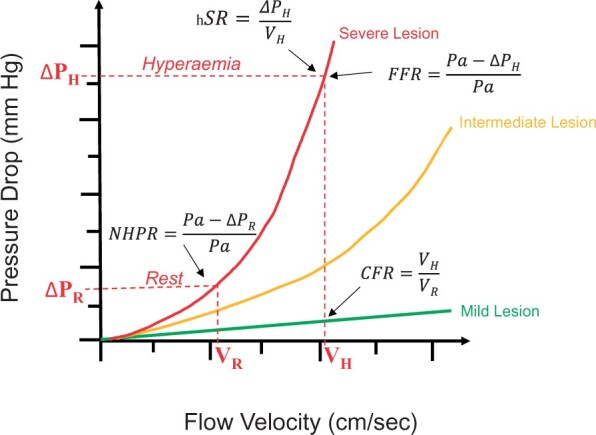
The relationship between flow velocity and pressure drop across a diseased coronary segment is unique to its severity and morphology. The figure depicts the curves across three illustrative stenoses of differing grades of severity and shows how this relationship forms the basis of all invasive physiological parameters used in clinical practice. CFR, coronary flow reserve; FFR, fractional flow reserve; hSR, hyperaemic stenosis resistance; NHPR, non-hyperaemic pressure ratio; P_H_, hyperaemic pressure; P_R_, resting pressure; V_H_, hyperaemic velocity; V_R_, resting velocity.

**Figure 3:**
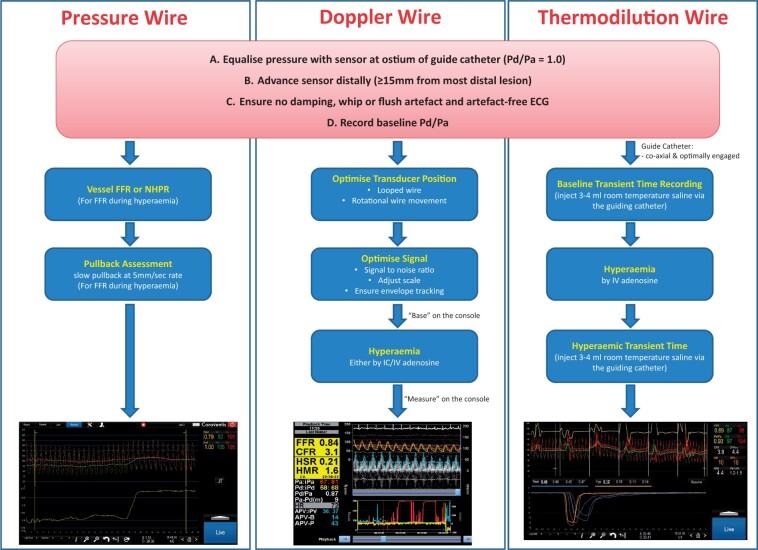
Guide to performing coronary physiology measurements using pressure, Doppler, and thermodilution wires. ECG, electrocardiogram; FFR, fractional flow reserve; IC, intracoronary; IV, intravenous; NHPR, non-hyperaemic pressure ratio; Pd/Pa, resting distal to aortic pressure ratio.

Autoregulation is the innate capacity of the coronary circulation to maintain stable flow across a range of perfusion pressures. Seminal canine experiments by Gould demonstrated that in the presence of an epicardial stenosis, resting flow remains constant until relatively severe stenoses, whilst maximal or hyperaemic flow diminishes with less severe coronary obstruction (*Figure 2*).^6^ In health, coronary blood flow increases roughly 3- or 4-fold with maximal demand and is expressed as the coronary flow reserve (CFR), the ratio of hyperaemic to resting flow in a vascular bed (*Table 1*). Whilst a diminished CFR may indicate ischaemia during periods of increased demand, such as exercise, it does not discriminate the location of impairment within the coronary vascular tree, which could be due to an epicardial stenosis, microvascular dysfunction, or both. When used to assess the combined significance of epicardial and microvascular disease, a threshold of 2.0 defines a circulation capable of causing ischaemia. However, for the purpose of selectively measuring functional epicardial stenosis severity, CFR has been largely replaced by indices of relative vascular conductance (i.e. indices that report on the effect of the stenosis compared to a situation in which that stenosis would be non-existing) based on coronary pressure measurements, which are discussed below. Furthermore, the reliability and accuracy of Doppler flow measurements depends on obtaining a stable transducer position within the coronary artery (*Figure 3*), which is associated with a learning curve and hence the technique is often limited to centres and operators with specific expertise.

**Table 1:** Coronary physiology indices

Index	Measures	Modality	Threshold	Notes
FFR	Epicardial stenosis severity	Pressure	≤0.80	Validated in large population Class I indication in ESC and ACC/AHA guideline Cost effective
NHPR	Epicardial stenosis severity	Pressure	≤0.89	Does not require pharmacological hyperaemia iFR—Class I indication in ESC and ACC/AHA guideline
hSR	Epicardial stenosis severity	Pressure and Doppler	≥0.80 Hg/cm s	Low variability Stenosis specific in presence of serial or complex coronary artery disease, and left ventricular dysfunction
CFR	Epicardial stenosis severity and microvascular resistance	Doppler/thermodilution and pressure	<2.5	In isolation does not distinguish between epicardial and microvascular compartments
hMR	Microvascular resistance	Doppler	>2.5	Pressure and flow velocity based Independent of stenosis severity
IMR	Microvascular resistance	Pressure and thermodilution	≥25	Pressure and thermodilution derived flow based Independent of stenosis severity Validated in CCS and ACS

ACC/AHA, American College of Cardiology/American Heart Association; ACS, actue coronary syndrome CCS, chronic coronary syndrome; CFR, coronary flow reserve; FFR, fractional flow reserve; hMR, hyperaemic microvascular resistance; hSR, hyperaemic stenosis resistance; IMR, index of microvascular resistance; iFR, instantaneous flow reserve; NHPR, non-hyperaemic pressure ratio.

The hyperaemic index of stenosis resistance (hSR) is the ratio of the pressure gradient across a coronary segment and average peak flow velocity measured during maximal hyperaemia [hSR = (pressure proximal to stenosis – pressure distal to stenosis)/average peak hyperaemic flow velocity]. The main advantage of hSR is that it selectively measures the resistance of an epicardial stenosis, independent of the state of the microvasculature. It has been shown to correlate better with reversible perfusion defects on single-photon emission computed tomography (SPECT) compared to traditional haemodynamic indices that are based solely on one modality, such as flow velocity (CFR) or pressure (FFR).^7^ ^,^ ^8^ An unobstructed coronary artery (without a pressure gradient) will have a value of 0 whilst the optimum threshold for detecting a haemodynamically significant lesion has been shown to be 0.80 mmHg/cm s. Combined use of intracoronary flow and pressure measurements may also circumvent limitations of flow-only- and pressure-only-based indices. Hence, in centres that have routine access and the requisite expertise, hSR may provide incremental information in situations where FFR may be misleading, such as elevated or variable microvascular resistance or when assessing the significance of a left main coronary lesion in the presence of downstream disease in one or both daughter branches. However, due to limited adoption of combined pressure and flow measurements in the catheterization laboratory, routine measurement of hSR has been largely confined to the research arena. As a consequence, indices based on flow and pressure measurements lack randomized outcome data, compared to the simpler measures below.

## Fractional flow reserve

In routine clinical practice, the assessment of coronary pressure is easier and less prone to error and variation compared to coronary flow. In 1975, David Young demonstrated the linear relationship between pressure and diameter stenosis during vasodilator-mediated hyperaemia (*Table 2*) by demonstrating pressure drop across an artificial stenosis created in canine femoral arteries.^9^ The simplification of these mathematical formulae and introduction of pressure sensor-tipped coronary guide wires heralded a new era of coronary physiology. In 1993, Pijls *et al.* demonstrated the utility of FFR in the coronary circulation of dogs wherein he described the theoretical basis for the calculation of CFR from coronary pressure—that the ratio of distal coronary to aortic pressure during maximal hyperaemia in a stenosed coronary artery was linearly and strongly correlated to flow in the diseased artery in relation to hypothetical flow in an entirely disease free artery supplying the same myocardial territory.^10^ Derivation of the simplified contemporary FFR equation has necessitated the following assumptions: (i) flow is linearly related to pressure; (ii) venous pressure (P_v_) is negligible and it is equal to zero flow pressure; and (iii) microvascular resistance always becomes constant and negligible during pharmacological hyperaemia.

**Table 2:** Coronary vasoactive drugs

Drug	Dose	Hyperaemia	Half life	Side effects	Advantage/disadvantages
IV adenosine	140 μg/kg/min	1–2 min	1–10 s	AV block Chest tightness Bronchospasm	Steady state hyperaemia Can be used for ostial lesions Pullback assessment of serial and diffuse disease- May be poorly tolerated by some patients Time taken for repeat measurements
IC adenosine	36–120 μg LCA 18–60 μg RCA	3–8 s	1–10 s	AV block	Well tolerated Quick repeat measurements Unable to do pullback Unable to interrogate ostial lesions
IC papaverine	10 mg LCA 15 mg RCA	30–60 s	2 min	QT prolongation Ventricular tachycardia Long half-life Lower blood pressure	Can be used in patients with asthma and COPD Safety profile not as favourable as adenosine

AV, atrioventricular; COPD, chronic obstructive pulmonary disease; IC, intracoronary; IV, intravenous; LCA, left coronary artery; RCA, right coronary artery.

In summary, FFR is an index of relative epicardial vessel conductance (i.e. compares with a vessel without stenosis and a hyperaemic Pd/Pa = 1), a fact that explains that its cut-off value (threshold for flow-limiting stenosis to guide revascularization ≤0.80) is the same as the Doppler-derived relative CFR (that uses a non-obstructed vessel as a reference) and its good correlation with relative CFR derived from PET.^11^ ^,^ ^12^ Like other pressure-derived indices, FFR is based on a very simplified model of coronary physiology, with numerous assumptions that may not always be fulfilled in some clinical scenarios. Yet, its introduction in clinical practice, facilitated by its simplicity, contributed enormously to a major advance in the functional evaluation of coronary stenoses. Numerous randomized controlled studies demonstrated that, compared with angiographic guidance, a FFR-guided revascularization strategy in chronic coronary syndromes results in a lower amount of revascularization and fewer major cardiovascular adverse events at follow-up (primarily driven by the reduced need for subsequent revascularization).^13^ ^,^ ^14^ Consequently, FFR is often regarded as the ‘gold-standard’ invasive coronary physiological index and used as the reference against which novel invasive and non-invasive ischaemic indices are compared, which belies the historical path by which FFR itself was validated, against existing non-invasive functional tests.^15^ FFR was the first physiological index to obtain a Class IA recommendation in international revascularization guidelines for evaluation of angiographically intermediate lesions.^13^ ^,^ ^14^ Recently, US Veterans Affairs data has revealed that the rate of FFR use has gradually increased from 14.8% to 18.5% among all patients with intermediate lesions between 2009 and 2017.^16^ Despite the widespread adoption of FFR by the interventional cardiology community, several features of FFR merit further appraisal.

To enable broad clinical application, an optimal dichotomous FFR threshold for detection of ischaemia was established. Using an FFR threshold of ≤0.80, there was no difference in rates of mortality or myocardial infarction at 5-year follow-up in the FAME-2 population between percutaneous coronary intervention (PCI) and medical-therapy groups.^5^ However, there were significantly greater rates of urgent revascularization in the medical-therapy arm, compared to the PCI arm, although the latter is highly likely to have been influenced by unblinded study design. Importantly, it is also worth appreciating that FFR reflects a risk continuum whereby there is an inverse relationship between FFR values and risk of adverse clinical event, raising the potential utility of FFR beyond merely a binary index. This is supported by a large patient-level pooled meta-analysis demonstrating that clinical events increased as FFR decreased and that revascularization showed larger net benefit for lower baseline FFR values.^17^ For the evaluation of FFR as a continuous variable, across its whole spectrum of values, statistical modelling has suggested that the optimal prognostic FFR to guide revascularization might be 0.67, which is notably similar to FFR values that correspond to flow impairment and myocardial ischaemia.^17^ ^,^ ^18^ This risk continuum observation was corroborated by the IRIS-FFR registry, that included >8,000 lesions, whereby statistical modelling suggested the optimal FFR threshold for cardiac death or myocardial infarction was 0.64.^19^

Low residual FFR values following PCI are associated with poorer patient outcomes.^17^ However, this scenario has a range of causes, including a sub-optimal result in the treated lesion and under-appreciated serial or diffuse CAD, which is present in one-third of patients undergoing coronary angiography.^20^ Therefore, prospectively predicting individual stenosis severity, when it is found in combination with other focal or diffusely diseased coronary segments is desirable. However, defining the impact of individual stenoses is hindered by haemodynamic interplay between serial lesions when using pressure-derived indices.^8^ Various strategies have been proposed to account for this interplay, including a method that requires measurement of coronary wedge pressure and hence has limited prospective utility.^21^ More recently a simplified mathematical correction has been proposed that incorporates routine pressure-wire pullback data, enabling isolation of individual stenosis physiologically using FFR.^22^ Another approach is direct quantitative evaluation of a hyperaemic pullback, based on the magnitude of pressure losses and the extent of functional disease, with lower pullback gradients indicating more diffuse coronary atherosclerosis.^23^

## Haemodynamic significance or vulnerable plaque?

In patients with stable CAD, there is little evidence to support revascularization of haemodynamically insignificant lesions. However, following acute coronary syndromes, there is debate about the relevance of potentially vulnerable plaques that do not cause appreciable luminal obstruction.^24^ ^,^ ^25^ This stems from a few small retrospective studies where coronary angiography was performed at varying time intervals from acute myocardial infarction suggesting that mild and moderate lesions were major contributors.^26^ ^,^ ^27^ Subsequent prospective studies, where more robust systemic evaluation was conducted, revealed that the majority of culprit lesions were in fact angiographically severe – demonstrated both angiographically and on intravascular ultrasound (IVUS).^26^ In the PROSPECT-I (Providing Regional Observations to Study Predictors of Events in the Coronary Tree) study, large plaque burden (≥70%), small minimal lumen area (≤4.0 mm²), or both, assessed by IVUS, identified angiographically mild lesions that were at increased risk of causing future coronary events, replicated by a few other studies.^28^ ^–^ ^30^ The more recent PROSPECT-II study identified non-culprit lesions by IVUS and their lipid content was assessed by near-infrared spectroscopy (NIRS). In this study of 3,629 non-culprit lesions in 898 patients, adverse events (cardiac death, myocardial infarction, unstable angina, or progressive angina either requiring revascularization or with rapid lesion progression) within 4 years occurred in 112 of 898 patients, with 66 arising from 78 untreated non-culprit lesions (mean baseline angiographic diameter stenosis 47%). Lesions with both large plaque burden by IVUS and large lipid-rich cores by NIRS had a 4-year major adverse cardiac event rate of 7.0% (95% confidence interval 4.0–10.0).^31^ These studies have demonstrated the clinical utility of identifying high-risk features in coronary imaging; however, how clinicians treat these lesions remains unclear. Furthermore, coronary angiography was used to adjudicate non-culprit lesion severity rather than FFR. Whether a combined algorithm that incorporates coronary physiological assessment and plaque characterization yields a better result may be the focus of future studies.

## Non-hyperaemic pressure-based indices

**Figure 4:**
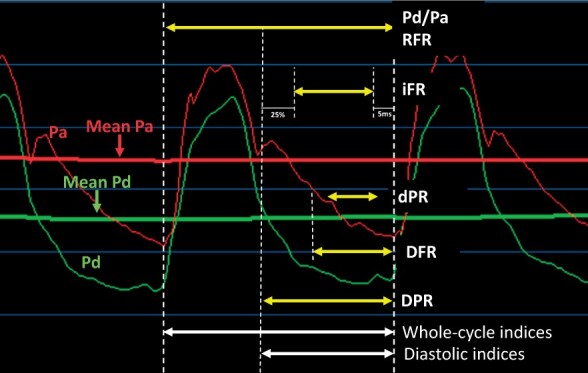
Illustration of different phases of cardiac cycle for evaluating non-hyperaemic indices. Pd/Pa (resting average distal pressure [Pd] divided by average aortic pressure [Pa]) is the average Pd/Pa during the entire cardiac cycle. Instantaneous wave-free ratio is the average Pd/Pa during the latter 75% of diastole, excluding the terminal 5 ms. Diastolic pressure ratio is the average Pd/Pa during the entire diastole. Diastolic pressure ratio is Pd/Pa during the wave-free period. Resting full-cycle ratio is the lowest mean Pd/Pa ratio during the entire cardiac cycle. Diastolic hyperaemia-free ratio is the average Pd/Pa during the period between Pa less than mean Pa ending at systole.

Over the last decade, there has been significant interest in non-hyperaemic or ‘resting’, pressure-derived coronary indices. The rationale for developing these indices included decreased procedural time and avoidance of the cost and side effects associated with the use of hyperaemic agents. Each non-hyperaemic index is a measure of Pd/Pa in a pre-specified window of the cardiac cycle (*Figure 4*).

Instantaneous wave-free ratio was introduced in 2012 and is based on Pd/Pa during the latter 75% of diastole, excluding the terminal 5 ms, an interval referred to as the ‘wave-free’ period, during which microvascular resistance is theoretically lowest in the cardiac cycle.^15^ Instantaneous wave-free ratio (iFR) and FFR are concordant in ∼80% of cases, using an iFR threshold of ≤0.89 and a reference FFR value of ≤0.80,^32–34^ and head-to-head comparisons against PET and myocardial perfusion imaging have shown comparable performance.^12^ ^,^ ^35^ ^,^ ^36^ Two large concurrent randomized controlled trials (and a pooled patient-level analysis of these same trials) demonstrated that revascularization based on an iFR at a threshold of 0.89 was non-inferior to revascularization based on a FFR threshold of 0.80 for the composite of death from any cause, non-fatal myocardial infarction, or unplanned revascularization; the overall event rate in this population was 6.1% to 7% at 12 months.^37^ ^,^ ^38^ Accordingly, international revascularization guidelines have since recommended that either iFR or FFR can be utilized for physiological stenosis assessment.^13^ ^,^ ^14^ Recently, a plethora of other non-hyperaemic indices have been described, including diastolic indices such as the diastolic hyperaemia-free ratio (DFR), diastolic pressure ratio (dPR), and diastolic pressure ratio (DPR) or whole cardiac cycle indices such as resting Pd/Pa ratio and resting full-cycle ratio (RFR).^39^ These have been shown to have excellent correlation with iFR suggesting that any non-hyperaemic index, regardless of the phase of the cardiac cycle on which each is based, can be utilized clinically, as they show excellent inter-index correlation.^39^ All non-hyperaemic pressure indices during the diastolic phase and RFR have the same threshold as iFR of 0.89. Head-to-head comparisons with PET suggest that they have the same diagnostic yield as FFR and iFR to diagnose ischaemia, although prospective prognostic data are still lacking.^40^

Despite increasing adoption, largely driven by the perception of ease, there remain several theoretical considerations that are pertinent to all non-hyperaemic indices. First, given the effect of neurohormonal variations on resting coronary flow, particularly under the often stressful conditions of the cardiac catherization laboratory, it may be expected that ‘resting’ coronary haemodynamics vary in a heterogeneous manner. Indeed, iFR estimation has been shown to be prone to variations in haemodynamic conditions that affect baseline coronary flow.^35^ In contrast, hyperaemic flow (and hence FFR) may be expected to be less affected by those neurohormonal effects. Second, it is important to take note that FFR itself, against which most of these indices have been validated, is an imperfect surrogate for ischaemia.^41^ In fact, this discordance has been demonstrated to have minimal implications for clinical outcome, although few of these studies have had adequate power to detect important but infrequently occurring cardiac events.^42^ ^,^ ^43^ Finally, as with FFR, the dichotomous iFR threshold has progressively migrated upwards from the initial derivation studies,^15^ from 0.83 in the ADVISE study to 0.86 in the CLARIFY study and 0.89 in DEFINE-FLAIR and iFR SWEDEHEART.^15^ ^,^ ^37^ ^,^ ^38^ ^,^ ^44^ This was illustrated in the SYNTAX II trial (prior to DEFINE-FLAIR results), which applied a protocol of physiology-based revascularization, which acknowledged the iFR grey-zone of 0.86–0.93 (with values within this range clarified by the administration of adenosine to calculate FFR) in patients with triple vessel disease.^45^ Interestingly, when using hSR as the physiological reference standard, different groups have found the optimal iFR value to be 0.86.^44^ ^,^ ^46^ This is akin to the FFR threshold being set at 0.75 in early studies and subsequent migration to 0.80.

## Angiographic estimation of fractional flow reserve

Angiographic simulation of coronary haemodynamics was introduced over four decades ago and recently has gained popularity in an attempt to avoid instrumentation of the coronary artery.^47^ At present. there are numerous angiography-based indices that are been utilized, including but not limited to: (i) quantitative flow ratio (QFR);^48^ (ii) vessel FFR;^49^ and (iii) FFR_angio_.^50^ These are all derived from three-dimensional reconstructions of the coronary tree via combining multiple angiographic projections. In addition, coronary haemodynamics are calculated using computational fluid dynamics based on the concept of a TIMI frame count, with calculations carried out by proprietary methods owned by each vendor. As with non-hyperaemic pressure-based indices above, the angiographic indices have been validated by comparison with FFR, as the ‘gold standard’ test; however, FFR has inherent limitations as outlined above. Thus far, QFR has the biggest evidence based supporting these indices; the FAVOR series of studies (Pilot, FAVOR II, and FAVOR II China) demonstrated the feasibility of carrying out these calculations and, in selected cases, demonstrated the superiority of QFR over three-dimensional quantitative coronary angiography in predicting the FFR value.^48^ ^,^ ^51^ ^,^ ^52^ Notwithstanding the appeal of a minimally invasive method of adjudicating stenosis severity, several limitations need to be acknowledged. First, three-dimensional vessel reconstructions are reliant on acquisition of optimal angiographic views to enable accurate luminal identification. This can be hampered by vessel tortuosity, ostial lesions, and overlapping vessels. In addition, stenosis assessment requires a reference vessel to provide lesion estimation; however, in ostial stenosis with no reference vessel, this may not be possible and/or fraught with erroneous calculations. Second, the use of TIMI count and/or computational fluid dynamics that underlie these indices is prone to significant error as greater assumptions are made in an effort to simulate coronary flow to derive physiological/haemodynamic stenosis quantification. Coronary flow is simulated using mathematical modelling; in addition to limitations outlined above, this is hampered by resting coronary haemodynamics as outlined for non-hyperaemic invasive indices (e.g. iFR), and magnitude and amount of contrast administered for opacification of the coronary tree. Currently, there are no randomized data supporting the safety of decision-making based on angiographic physiological stenosis assessment, compared with intracoronary indices.

## Non-invasive estimation of fractional flow reserve

Over the last decade, the role of computed tomography coronary angiography (CTCA) has evolved from a test to rule out the presence of CAD in low-risk populations to being considered an appropriate first-line test in patients with new-onset angina, almost regardless of their pre-test likelihood of CAD.^13^ However, the ability to predict physiologically significant CAD by CTCA is limited.^53^ CT-FFR applies computational flow dynamic modelling to simulate FFR. This requires inflow and outflow modelling of the coronary vasculature with simulated hyperaemia; the latter requires artifact-free coronary artery images and additional calculations of cardiac output, aortic pressure, and microvascular resistance.^54^ Of note, calculation of CT-FFR does not require intravenous adenosine administration, although it is usually performed after administration of nitrates. CT-FFR improves diagnostic accuracy compared to anatomical CTCA alone, with accuracy in the range of 81–97%, depending on the population enrolled.^55^ CT-FFR has been reported to have better patient and vessel-level diagnostic accuracy than SPECT but inferior patient-level diagnostic performance compared to PET.^56^ The ability to use CT-FFR to predict individual lesion significance in serially diffused coronary arteries is limited at present, although the CT-FFR virtual planning tool has promise, even though it is not possible to obtain real-time procedural information at present.^46^ The assumptions inherent in the computational fluid dynamic modelling may explain the inaccuracies of CT-FFR that are sometimes encountered, including assumptions on the amount and viability of subtended myocardium and the inability to characterize the variable contribution of microvascular resistance. CT-FFR is also reliant on high-quality imaging and a proportion of CTCAs may be unsuitable for CT-FFR analysis—interpretation and diagnostic accuracy of both CTCA and CT-FFR are adversely impacted by numerous factors including coronary calcium and motion artefact. However, the use of dual-source technology and improved imaging algorithms have decreased the rejection rate.^57^

## Non-invasive assessment of myocardial perfusion

Vasodilator-stress myocardial perfusion imaging with SPECT, PET, CMR, and most recently computed tomography (CT) allows the detection of inducible perfusion defects and the diagnosis and risk stratification of patients with known or suspected CAD. Although using different tracers and detectors, the basic principles of the four modalities are similar, involving peripheral injection of a method-specific contrast agent followed by the acquisition of a dynamic series of images tracking the myocardial contrast passage. Images are typically acquired at rest and during vasodilator, or sometimes physical or inotropic, stress. Interpretation in clinical practice remains most commonly visual, and perfusion defects are identified as regions with relatively reduced myocardial signal increase. In addition, modelling of the contrast kinetics in particular of dynamic PET and CMR perfusion data allows quantitative estimates of myocardial blood flow (MBF) to be derived. Visual analysis of myocardial perfusion imaging yields high diagnostic accuracy for the detection of both anatomically and FFR-defined coronary stenosis,^58^ ^,^ ^59^ with areas under the receiver operating characteristic curve of 0.82, 0.94, 0.93, and 0.94 at the patient level for SPECT, CMR, PET, and CT, respectively. A limitation of quantitative myocardial perfusion remains that cut-offs for the diagnosis of CAD vary between studies and modalities and no uniform threshold has been established. As with CFR, myocardial perfusion at the tissue level is a composite of epicardial and small vessel function, which is both a limitation and a strength. This may partly explain why FFR and non-invasive indices have been found to be discordant in up to 40% of cases.^60^ In the absence of epicardial coronary disease, abnormal stress MBF or MBF reserve is considered to reflect microvascular dysfunction and is strongly associated with adverse cardiovascular outcome.^61^ Different thresholds have been proposed for the detection on microvascular disease with non-invasive myocardial perfusion imaging, typically ranging from 1.4 to 2.2 for myocardial perfusion reserve, varying with imaging modality, study population and invasive comparator. In a direct comparison of invasive Doppler-derived CFR, myocardial perfusion reserve from high-resolution CMR showed an area under the curve of 0.88 at a threshold of 2.19 for the detection of coronary microvascular dysfunction (CMD) in a study of 75 patients. Visual analysis of CMR and stress or rest perfusion alone were less accurate but improved when using the endocardial to epicardial ratio stress perfusion ratio, taking advantage of the high spatial resolution of CMR.^62^ ^63^

## Physiology testing to predict and optimize the outcome of PCI

Over the last two decades, the majority of coronary physiology studies have focused on their role and accuracy as diagnostic tests. However, recent interest has centred on the use of these same indices to evaluate the quality and adequacy of PCI. The DEFINE-PCI study found that blinded post-PCI physiological pullback assessment detected residual substrate for ischaemia in around 25% of patients after coronary stenting, despite the procedure been completed due to an operator-determined angiographically successful result.^20^ Of note, among patients with suboptimal post-PCI physiology, 82% had untreated focal stenoses that were angiographically inapparent, of which 38% were located within the stented segment. Similarly, the TARGET FFR study demonstrated residual ischaemia post-PCI in 29% of patients. In addition, based on pullback assessment, further optimization was peformed in 30.5% of the patients ramomized to physiology-guided optimzation stratery arm.^64^ Suboptimal post-PCI physiology can be attributable to numerous factors including sub-optimal stent result, underappreciated impact of pre-existing concomitant lesion(s) or presence of diffuse disease. In most cases, the relative physiological contribution of diffuse or focally diseased segments is estimated by carrying out a slow manual pullback of the pressure wire in the artery, with the rate and magnitude of change of each index evaluated visually. However, both resting and hyperaemic pullback can underestimate the physiological severity of individual stenosis in the presence of serial disease and it has recently been shown that the resultant errors can be ameliorated with the utilization of mathematical correction models.^22^ Importantly, clinical outcome studies are required, for these novel techniques, to determine their respective application in serial stenosis.

## Ischaemia with unobstructed coronary arteries

Nearly half of all patients with angina have non-obstructive coronary artery disease (NOCAD), comprising a range of pathophysiological diagnoses—including microvascular smooth muscle or endothelial dysfunction or coronary vasospasm, which culminate in abnormalities of CFR.^65^ In the context of NOCAD, a diminished CFR, synonymous with CMD, correlates with a greater risk of heart attacks, strokes and death.^66^

**Figure 5:**
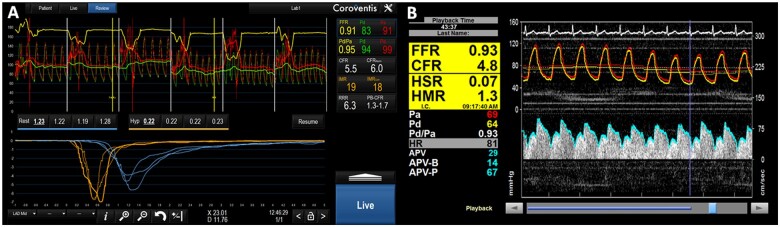
Coronary haemodynamic evaluation with dual sensor coronary wire. (*A*) Coronary pressure and thermodilution. (*B*) Coronary pressure and Doppler.

**Figure 6:**
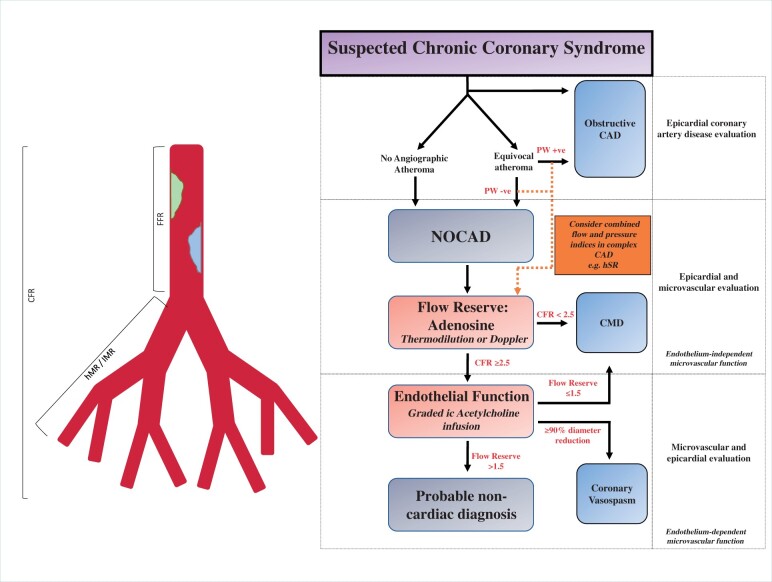
Comprehensive assessment of suspected chronic coronary syndrome. Complex coronary artery disease refers to serial stenoses, left main stem stenosis, or coronary artery disease in the presence of impaired left ventricular function. Under these circumstances, utilization of combined flow and pressure-derived physiology indices can assist tailored management strategy. Patients with substrate suggestive of NOCAD should be considered for endothelium-independent microvascular function assessment by coronary flow measurement(s). CFR, coronary flow reserve; CMD, coronary microvascular dysfunction; FFR, fractional flow reserve, hMR, hyperaemic microvascular resistance; hSR, hyperaemic stenosis resistance; ic, intracoronary; IMR, index of microvascular resistance; NOCAD, non-obstructive coronary artery disease, PW, pressure wire.

Stratifying treatment in patients with NOCAD based upon CFR values yields superior outcomes to empirical therapy, supporting the role of ad hoc coronary physiology testing in this large patient population.^67^ ^,^ ^68^ Measurement of pressure and flow allows calculation of microvascular resistance and there is emerging evidence that minimal microvascular resistance [measured by the Doppler-derived hyperaemic microvascular resistance (hMR) or the thermodilution-derived index of microvascular resistance (IMR)] can be used to further classify patients with CMD into distinct ‘structural’ (low CFR, high IMR, or hMR) and ‘functional’ (low CFR, normal hMR, or IMR) endotypes, which have similar phenotypes but distinct underlying pathophysiological process that may in turn represent therapeutic targets in the future (*Figure 5*).^69^ More advanced ‘structural CMD’ endotypes are associated more commonly with heart attack and deaths, whilst ‘functional CMD’ is associated with chest pain hospitalizations. As our understanding of the pathophysiological processes leading to CMD, therapy will likely be stratified based on detailed endotypes.^69^ CMR imaging has recently shown promise in the detection of CMD among stable angina populations using semi-quantitative analysis of hyperaemic MBF.^70^ Transmural maldistribution of MBF during hyperaemia is a hallmark of CMD, and high-resolution perfusion CMR with quantification of MBF across myocardial layers has greater accuracy at identifying this condition.^71^ Higher-throughput sequences increasingly forego acquisition of rest perfusion images and rely upon visual assessment alone in the interest of time, and whilst this approach is acceptable when ruling out obstructive CAD, this approach has been shown to drastically reduce the accuracy of identifying CMD.^63^ ^71^ ^72^ Nevertheless, among patients who have persistent refractory symptoms despite a normal CTCA and perfusion CMR, coronary reactivity testing may be considered, as coronary vasospasm and endothelial dysfunction currently remain invasive diagnoses. In NOCAD, pressure-only surrogates of flow are not applicable, as there is no appreciable pressure drop across the course of an unobstructed artery. Whilst there is interest in the possible role of corrected TIMI frame count, an index of coronary flow as a continuous quantitative variable, the number of cine frames required for contrast to first reach standardized distal coronary landmarks in the infarct-related artery, in identifying CMD, this approach has not been validated when comparing to the invasive reference standard. In summary in patients with NOCAD, assessment of flow during adenosine administration would be a minimum perquisite to ruling out CMD, with additional use of acetylcholine enhancing sensitivity, whilst resistance measurements may inform about the degree of systemic disease involvement, underlying pathophysiology and prognosis (*Figure 6*). The adoption of comprehensive coronary algorithms may be hampered by the additional time required but may reduce repeat admissions to acute cardiovascular services. Moreover, establishing a diagnosis facilitates tailored patient management. When assessing microvascular dysfunction, vasoactive medications should ideally be withheld for at least 24 h beforehand, if possible, to optimize diagnostic yield.

## Conclusions

The last two decades have seen technological developments which have progressively resulted in techniques that can be readily applied in most cardiac catheter laboratories to enable detailed stenosis evaluation. The advent of commercially available dual pressure and Doppler/thermodilution flow wires provide the basis to perform assessment of both epicardial and microvascular compartments with relative ease and speed. The introduction of pressure-based estimations of flow has revolutionized the field and brought physiology into mainstream clinical use. However, uptake has been suboptimal and, in a bid to increase this, new indices have been proposed that do not require induction of hyperaemia, one of the key conditions considered necessary to estimate flow using pressure measurements alone. The latest generation of tests aims to use just angiographic data to estimate coronary flow and stenosis severity.

However, each iterative simplification comes at the cost of potential limitations, principally due to assumptions inherent in the techniques, which may affect clinical accuracy. To minimize diagnostic ambiguity in patients presenting with chest pain, a stepwise approach should be adopted to optimize the assessment of epicardial disease. This should incorporate algorithmic escalation to more accurate, but perhaps less convenient, techniques should the real-time results of the simpler measures yield equivocal results. Assessment of microvascular and endothelial function should be considered in patients with angina and/or ischaemia in the absence of epicardial disease by simultaneously measuring coronary flow and pressure.

## Funding

This work was supported by the British Heart Foundation [PG/19/9/34228 and FS/16/49/32320] and the National Institute for Health Research via the Biomedical Research Centre award to King’s College London.

**Conflicts of interest:** T.P.v.d.H. is a consultant and a speaker at educational events for Abbott and Philips. J.E. is a consultant and a speaker at educational events for Abbott, Boston Scientific, and Philips. J.J.P. is a consultant for Philips. None of the other authors have any conflict of interest or relationships with industry that could have influenced this manuscript.
